# Drug repurposing against COVID-19: focus on anticancer agents

**DOI:** 10.1186/s13046-020-01590-2

**Published:** 2020-05-12

**Authors:** Gennaro Ciliberto, Rita Mancini, Marco G. Paggi

**Affiliations:** 1 Scientific Director, IRCCS – Regina Elena National Cancer Institute, Rome, Italy; 2 Department of Clinical and Molecular Medicine, Sant’Andrea Hospital, Sapienza University of Rome, Rome, Italy; 3 Cellular Networks and Molecular Therapeutic Targets, Proteomics Unit, IRCCS – Regina Elena National Cancer Institute, Rome, Italy

**Keywords:** Health emergencies, Viral pneumonia, Anticancer drugs, Drug repurposing, Immune response, BCG

## Abstract

**Background:**

The very limited time allowed to face the COVID-19 pandemic poses a pressing challenge to find proper therapeutic approaches. However, synthesis and full investigation from preclinical studies to phase III trials of new medications is a time-consuming procedure, and not viable in a global emergency, such as the one we are facing.

**Main Body:**

Drug repurposing/repositioning, a strategy effectively employed in cancer treatment, can represent a valid alternative. Most drugs considered for repurposing/repositioning in the therapy of the COVID-19 outbreak are commercially available and their dosage and toxicity in humans is well known, due to years (or even decades) of clinical use. This can allow their fast-track evaluation in phase II–III clinical trials, or even within straightforward compassionate use.

Several drugs being re-considered for COVID-19 therapy are or have been used in cancer therapy. Indeed, virus-infected cells are pushed to enhance the synthesis of nucleic acids, protein and lipid synthesis and boost their energy metabolism, in order to comply to the “viral program”. Indeed, the same features are seen in cancer cells, making it likely that drugs interfering with specific cancer cell pathways may be effective as well in defeating viral replication.

**Short Conclusion:**

To our knowledge, cancer drugs potentially suitable for facing SARS-CoV-2 infection have not been carefully reviewed. We present here a comprehensive analysis of available information on potential candidate cancer drugs that can be repurposed for the treatment of COIVD-19.

## Background

The coronavirus disease 2019 (COVID-19), a mild-to-severe respiratory illness associated with symptoms (fever, cough and shortness of breath), is caused by severe acute respiratory syndrome coronavirus 2 (SARS-CoV-2). The very limited time allowed to face the COVID-19 pandemic, as well as its severity, imposes an unwavering commitment – from the scientific community – to find proper therapeutic approaches.

As for several infectious diseases, vaccination will likely able to generate a safe, long-lasting protection from SARS-CoV-2 infection, but this approach is not suitable for the current COVID-19 outbreak. As an alternative, antiviral drugs, or modulators of the host immune response can be considered. However, synthesis and full investigation - from preclinical studies to phase III trials - of new medications is a time-consuming procedure, and is not viable in a global emergency, such as this.

Conversely, drug repurposing/repositioning, a strategy effectively employed in cancer treatment [1–3], can represent a valid alternative, provided that suitable medications are selected among the enormous number of potential, already synthesized, and often already clinically employed, compounds.

Drug repurposing has already been suggested for specific drugs in the treatment of the current COVID-19 outbreak [4–9]. Most drugs considered for repurposing/repositioning in the therapy of the COVID-19 outbreak are commercially available and their dosage and toxicity in humans is well known, due to years (or even decades) of clinical use. This can allow their utilization in faster and less expensive phase II–III clinical trials, or even within straightforward compassionate use.

In particular, a remarkable number of drugs re-considered for COVID-19 therapy are or have been used in cancer therapy. This should not be surprising if we consider that virus-infected cells are pushed to enhance the synthesis of nucleic acids, protein and lipid, and boost their energy metabolism, in order to comply to the “viral program”. Indeed, the same features are seen in cancer cells, making it likely that drugs interfering with specific cancer cell pathways may be effective as well in defeating viral replication.

To our knowledge, cancer drugs potentially suitable for facing SARS-CoV-2 infection have not been exhaustively reviewed. In order to make a rational and effective choice of drugs amenable of repurposing for the therapy of COVID-19, we can elaborate existing data, from experimental and translational research, clinical trials, anecdotal reports and other published information.

We present here a comprehensive analysis of available information on potential candidate cancer drugs that can be repurposed for the treatment of COIVD-19.

## Main text

**Fig. 1:**
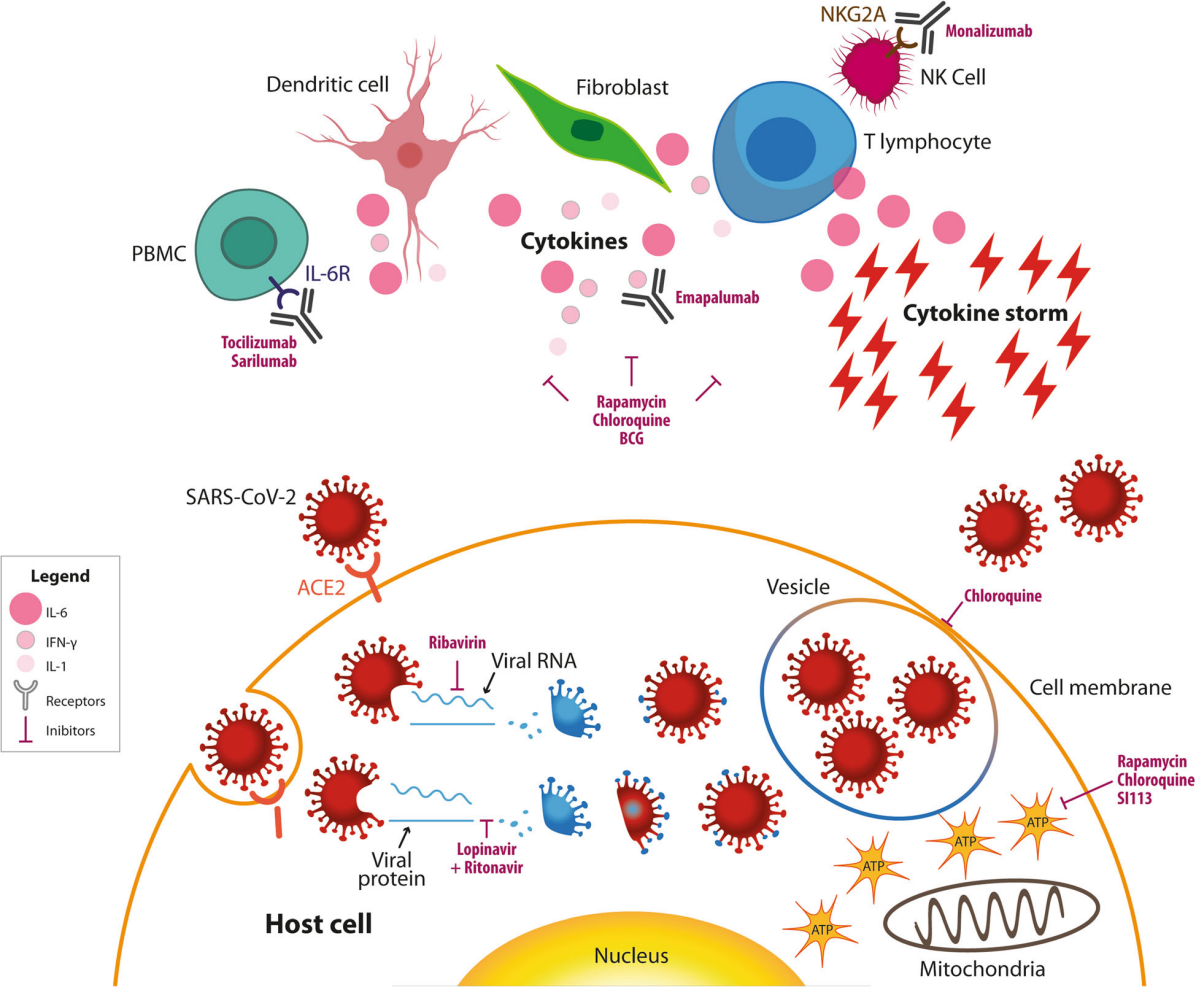
SARS-CoV-2 replication, host response and interfering drugs. The drugs able to counteract the processes underlying SARS-CoV-2 replication and consequent host response, and their sites of action are shown. PBMC = Periferal Blood Mononucleate Cells

Potentially suitable drugs for repositioning are essentially those affecting signal transduction, synthesis of macromolecules and/or bioenergetic pathways, those able to interfere with the host immune response, in particular, the life-threatening cytokine storm associated with severe COVID-19 and finally antiviral compounds are occasionally effective in fighting cancer (Table 1. Please note that research in the field is growing, and therefore the list may be incomplete at the time of publication). A single molecule can present more than one of the above-mentioned mechanisms. Drugs and their mechanism of action in relation to SARS-CoV-2 infection and host response are depicted in Fig. 1.

**Table 1:** Old and new drugs tested or used in the oncological setting and potentially useful in COVID-19 therapy

Drug	MoA
Rapamycin and derivatives	Immunosuppressant; PI3K/mTOR inhibitor; inhibitor of viral replication
Chloroquine and derivatives	Antimalarial; broad spectrum anti-infective agent; interferent with protein post-translational processes; autophagy inhibitor; MAPK inhibitor; inhibitor of pro-inflammatory cytokines
SI113	SGK1 inhibitor
Tocilizumab	MoAb targeting IL-6R, thus contrasting cytokine storm and fibrotic degeneration
Sarilumab	MoAb targeting IL-6R, thus contrasting cytokine storm and fibrotic degeneration
Emapalumab plus anakinra	MoAb targeting IFN-γ plus IL-1R antagonist
Monalizumab	MoAb targeting NKG2A
BCG	Tuberculosis prevention; inhibition of a TGF-β1-mediated EMT
Lopinavir plus ritonavir	Viral Protease inhibitors approved for HIV treatment
Ribavirin	Viral RNA synthesis inhibitor; RdRp inhibitor
Remdesivir^a^	Viral RNA polymerase inhibitor

*BCG* Bacillus Calmette-Guérin, *EMT* Epithelial-to-mesenchymal transition; *MoA* Mechanism of Action; *MoAb* Mono- clonal antibody; *RdRp* RNA-dependent RNA polymerase

^a^Not used in oncological settings

### Drugs interfering with signal transduction and/or bioenergetics pathways

#### Rapamycin and derivatives

Rapamycin (sirolimus) has a long history of repositioning. It was first used as an antifungal, followed by an immunosuppressive agent in organ-transplanted patients and, more recently, also as an anticancer agent [2, 10]. Its cellular target has been named mTOR (mammalian Target Of Rapamycin) after the name of the compound itself, showing specific activity toward the mTORC1 complex [11]. Rapamycin is also effective in the therapy of the autoimmune lymphoproliferative syndrome [12]. Rapamycin decreases mTOR phosphorylation (mTORC1) [13], causing a downstream perturbation of this signal transduction pathway. The consequent catabolic inhibition and ATP shortage evokes the activation of AMPK [14] and of its substrate ACACA, promoting autophagy, a physiological procedure through which cells recycle old organelles or damaged proteins in order to provide an alternative energy supply [15, 16].

mTOR inhibition counteracts viral replication and improves outcomes in patients infected by Andes virus [17], HCV [18], Middle-East respiratory syndrome coronavirus (MERS-CoV) [19] and H1N1 pneumonia [20]. In addition, rapamycin (alone or in combination with actinomycin D) has been recently proposed to interfere with the SARS-CoV-2 interactome in a systems pharmacology-based network medicine platform [5]. As specified above, rapamycin also presents immunosuppressant activity, which could be relevant in mitigating the SARS-CoV-2-induced inflammatory response.

There are some rapamycin derivatives available, e.g. temsirolimus, everolimus and ridaforolimus, which display slightly different pharmacokinetic characteristics and may be worth evaluation in the treatment for COVID-19. Among these, ridaforolimus has been tested in phase II clinical trial compared with progestin or investigator choice chemotherapy in advanced endometrial carcinoma showing encouraging results, but elevated toxicity, confirming the significance of the mTOR pathway in these neoplasms [21].

#### Chloroquine and hydroxychloroquine

Chloroquine (CQ) is a drug characterized by several decades of clinical use due to its well-renowned preventive and curative antimalarial activity. More recently, CQ has attracted the oncologists for its ability of interfering with the late stages of autophagy, by producing cytoplasmic accumulation of non-functional autophagic vesicles [22]. Both normal and cancer cells utilize autophagy for energetic purposes, but cancer cells, due to their higher energy requirements, rely more actively on autophagy, especially after being stressed by radio- or chemotherapy [23–25]. Therefore, the association of first-line therapeutic approaches in cancer patients with autophagy inhibitors has been largely investigated [26, 27] and suggested [26–28] and clinically investigated in clinical trials, mainly in CNS tumors [29–31].

CQ also possesses broad anti-infective and anti-viral properties [32], especially against flaviviruses, retroviruses and coronaviruses [33]. Indeed, CQ can interfere with sialic acid biosynthesis, compromising the post-translational modifications of the transmembrane viral binding proteins [34, 35], thus impairing viral penetration inside the cell. Indeed, interaction between SARS-CoV-2 and the membrane receptor angiotensin-converting enzyme 2 (ACE2), maximally expressed in lung alveolar epithelial cells, enterocytes of the small intestine, Leydig cells and Sertoli cells, strongly depends upon glycosylation [36]. CQ also induces alkalization of endosomes, thus inhibiting endocytosis of the viral particles and their enzymatic degradation by proteases [37–39], an essential step for the release of functional viral nucleic acid [33]. Furthermore, CQ improves viral antigen presentation and thus enhances T-cell-mediated immunity [40].

Another major advantage of CQ is its ability to modulate the inflammatory response and reduce the synthesis of pro-inflammatory cytokines. This molecule has been used since decades in the treatment of abnormal inflammatory responses (sarcoidosis) and autoimmune disorders (rheumatoid arthritis; lupus erythematosus). The ability of CQ to reduce cytokine immune response [41] could be functional in governing the cytokine storm associated with COVID-19 [42].

A mechanism similar to the one described for the inhibition of the autophagosomes could be speculated to impair formation and release of virion-containing vesicles by infected cells.

Very recently, CQ has been used in COVID-19 therapy at the dose of 500 mg/day with favorable results [39], butother studies demonstrate high toxicity and scarce effect of either CQ [43] or its analogue hydroxychloroquine (HCQ) [44, 45] in treating patients with severe COVID-19.

Since autophagy is regulated by the interplay between mTOR [46] and AMPK [15], the use of CQ in combination with drugs able to interfere with these pathways should be carefully evaluated.

CQ has a well-known safety profile, but it is associated with toxic retinopathy, renal and cardiac toxicity, which occur when the safe dose is exceeded. HCQ possesses slightly different pharmacokinetic properties and displays less overall toxicity. HCQ is commercially available worldwide, which it is not the case for CQ, whose distribution has been discontinued in some countries [47]. Both CQ and HCQ are being considered for use to preventing COVID-19 in SARS-CoV-2 post-exposure and long-term prophylaxis [48].

#### SI113

SI113 is a small molecule able to inhibit the activity of SGK1, an AKT-related kinase involved in the PI3K/mTOR pathway and in EMT [49]. This kinase plays a pivotal role in cancer proliferation and drug resistance [49, 50] and is sensitive to the small molecule SI113 [51], which is thus able to inhibit cancer cell growth in vitro and in vivo [52, 53] via a multifaceted mechanism of action, including inhibition of the PI3K/mTOR pathway and stimulation of autophagy [54].

Infection by MERS-CoV, a cognate of SARS-CoV-2, induces a massive inflammatory response, possibly related with fibrosis, mainly via the upregulation of the T helper (Th) 1 and Th17 cells [55]. Of note, IL-17-producing Th cells are induced by the activity of SGK1 [56]. Additionally, experimental models of inflammatory bowel disease showed the role of Th17 and SGK1 as mediators of the Th17 switch [57]. Therefore, SI113 could deserve evaluation in the prevention of the cytokine storm-induced lung fibrosis.

It should be noted that SI113 has never been used in humans, but it is effective in reducing tumor growth in cancer-bearing mice, appearing also well tolerated and non-toxic [52, 53]. Thus, SI113 cannot be considered a repurposed drug, although preclinical models indicate it as potentially effective in COVID-19 therapy. We included this drug in the present manuscript for the sake of completeness.

### Immunomodulatory medications

#### Tocilizumab

This compound is a humanized monoclonal antibody (MoAb) targeting interleukin-6 receptor (IL-6R). Pharmacology, pharmacokinetics, clinical efficacy, safety, and role of tocilizumab in rheumatoid arthritis (RA) are well-established [58] and possibly due also to its effect on the AKT/mTOR pathway [59]. Tocilizumab has also been approved for the treatment of the cytokine storm associated with cancer immunotherapy [60] or, more often, with CAR-T therapy [61, 62]. This MoAb does not have direct antiviral effects, but effectively contrasts the massive cytokine release syndrome displayed in severe COVID-19 by antagonizing the binding of IL-6, one of the cytokines most involved in this process, to its receptor [58]. After the first report of the effectiveness of tocilizumab in restraining the cytokine storm deriving from SARS-CoV-2 infection [63], this drug is currently under evaluation in a multicenter phase II clinical investigation in Italy on its efficacy and safety in patients with COVID-19 pneumonia [64]. The FDA has approved a phase III randomized, double-blind, placebo-controlled study to assess the effectiveness of tocilizumab in hospitalized patients with severe COVID-19 pneumonia [65]; in addition an increasing number of clinical trials involving the use of this drug in the treatment of COVID-19 are ongoing (https://clinicaltrials.gov/).

#### Sarilumab and Emapalumab

Sarilumab is an anti-IL-6Rα MoAb approved for moderate-to-severe rheumatoid arthritis [66] with a well-defined role also in blocking IL-6 action in cancer [67],while emapalumab, directed toward interferon (IFN)-γ, is used in the therapy of hemophagocytic lymphohistiocytosis [68, 69] and is employed in combination with anakinra, an IL-1R antagonist, in RA patients [70]. As tocilizumab, also sarilumab and emapalumab can effectively counteract the massive cytokine release related with SARS-CoV-2 infection. Agenzia Italiana del Farmaco (AIFA) approved the use of either sarilumab or emapalumab in phase II–III clinical studies involving hospitalized patients with COVID-19 pulmonary complications, with the aim to counteract the cytokine storm [64].

#### Monalizumab

Monalizumab is a MoAb directed toward NKG2A (CD94), a receptor for the recognition of MHC class I HLA-E molecules. NKG2A is gaining relevance as a key player in cancer-mediated immune checkpoint blockade and its neutralization by monalizumab restores the host immune response toward cancer [71]. Monalizumab is under clinical investigation in advanced gynecologic malignancies [72]. Interestingly, NKG2A appears overexpressed in cytotoxic T lymphocytes and natural killer cells in SARS-CoV-2-infected patients [73, 74], where it may reestablish the host immune response and increase survival in patients with severe pneumonia.

#### Bacillus Calmette-Guérin

Bacillus Calmette-Guérin (BCG), an invaluable tool for vaccination against tuberculosis, has been widely used as a concomitant therapeutic approach for lung cancer [75], and is considered as an overall protection from lung cancer incidence [76]. More recently, BCG has been successfully used for the local treatment of intermediate/high-risk bladder cancer [77].

BCG presents recognized immunomodulatory properties [78] and is associated with reduced risk of asthma [79]. Immunization via BCG provides relief from airway inflammation through the inhibition of a TGF-β1-mediated epithelial-to-mesenchymal transition (EMT), inhibiting the related remodeling of the respiratory tract accompanied with loss of lung epithelial integrity and fibrotic evolution [80]. In the COVID-19 setting, epithelial integrity of the respiratory tract is fundamental, since permanent lung fibrosis is a serious risk for severe and critically severe COVID-19 survivors [42]. Therefore, BCG might reduce the risk of severe disease progression and potentially reduce the mortality and disability rate.

### Antiviral compounds

Clearly, some of the drugs under consideration for repurposing for COVID-19 therapy are antiviral compounds, usually nucleoside analogues, i.e., small molecules mimicking ribonucleosides or deoxyribonucleosides able to inhibit viral replication after being incorporated within the viral nucleic acid sequence. These drugs have been used since decades as antivirals, although their clinical efficacy is often associated with the onset of drug resistance. Some antiviral drugs show interesting anticancer properties, being effective in inhibiting important signal transduction pathways, in vitro and in vivo [81, 82].

#### Lopinavir plus ritonavir

The association of the protease inhibitors lopinavir and ritonavir is an approved treatment for HIV treatment. It is effective in restraining the growth of urological malignancies in vitro, where induces endoplasmic reticulum stress, mTOR inactivation and AMPK boosting [83]. The same drug combination has been also evaluated in the treatment of cervical cancer patients [84].

The association between lopinavir and ritonavir is effective in reducing the risk of adverse clinical outcomes and viral load in SARS patients [85]. On these bases, this cocktail has been proposed for the treatment of COVID-19, but a very recent clinical trial showed no benefits in adult patients with severe disease [86].

#### Ribavirin

This drug is a guanosine analogue and RNA synthesis inhibitor successfully employed in the therapy chronic hepatitis C virus (HCV) infection [87]. As far as cancer is concerned, this compound induces GTP depletion in HeLa cervical cancer cells [88] and is effective in inhibiting glioblastoma growth in vitro and in vivo in preclinical models [81]. Along this line, the efficacy of ribavirin in the oncological setting is being investigated in ongoing clinical trials in acute myeloid leukemia, oropharyngeal squamous cell carcinoma, and breast cancer [89]. Ribavirin is also endowed with COVID-19 anti-RNA-dependent RNA polymerase (RdRp) activity [4]. Clinical trials are ongoing, based upon available data regarding dosage and toxicity derived from broad experience on the use of this drug as an anti- HCV compound.

#### Remdesivir

This compound is not cancer-related, but deserves to be mentioned as a paradigmatic case of effective repositioning. It is a prodrug of an adenine analogue, thus a viral RNA polymerase inhibitor, used during the Ebola outbreak [90]. Remdesivir has been found effective in vitro against SARS-CoV-2 infection when administered in concomitance with the antimalarial CQ (see above) [91] and in vivo in a primate model (rhesus macaque), either as prophylaxis or therapy of MERS-CoV infection [92]. Presently, clinical trials on remdesivir in COVID-19 are enrolling patients and are supported by the National Institutes of Health (NIH) [93], USA and AIFA, Italy [64]. Compassionate use of remdesivir in COVID-19 patients in a single-arm clinical trial gave positive preliminary outcomes [94], which appear in contrast with the results published by another group [95]. While the debate over the efficacy of this drug is still open, according to the preliminary results reported in the ACTT NIH clinical trial [93], the FDA has given remdesivir an emergency use authorization restricted to patients affected by severe COVID-19 [96].

## Conclusions

*COVID-19: a lesson to be learned.* The SARS-CoV-2 pandemic has been generated by a new strain of the coronavirus that has never previously been identified in humans. This virus is phylogenetically close to SARS-CoV, the causative agent of SARS. SARS-CoV-2, which reached humans via a spillover process from other animal species, possesses a peculiar tropism for the airway epithelium in humans, showing also elevated contagiousness and an extremely variable clinical course of its infection.

The COVID-19 outbreak found the world definitely unprepared to handle such a global emergency. Similar concerns must be raised toward a potential novel strain possibly responsible for future viral outbreaks, in order not to replicate the extremely negative outcome of the influenza A H1N1 1918–1919 “Spanish” pandemic [97]. To this end, it is mandatory to work prospectively to produce or identify better antiviral drugs and prophylactic/therapeutic MoAb therapies, as well as possibly targeting vital pathogenic factors, such as, in the case of SARS-CoV-2, Spike protein RBD [36, 98] or the main protease M^pro^ [99]. In addition, a study on the immune response of patients that have recovered from SARS-CoV-2 infection could be of great interest, in line with what was carried out for the Ebola survivors [100].

The forcedly limited number of drugs briefly described in this review appear to act essentially through selected mechanisms, i.e., a) inhibition of the PI3K/AKT–SGK1/mTOR signaling cascade; b) inhibition of the cytokine storm; and c) inhibition of viral nucleic acid synthesis. The activation of the PI3K/AKT–SGK1/mTOR pathway appears fundamental for supporting the replication of various virus species in the host [17–20] by boosting their energy metabolism and reactive oxygen species production, especially in the cells of the immune system [101, 102]. Therefore, drugs able to interfere with mTORC1 signaling can produce ATP shortage in the cells in which the virus is replicating, characterized by an excess of energy requirements. Such a metabolic pattern is reminiscent of the peculiar setup of the energy metabolism in cancer cells, i.e. Warburg effect [103, 104], where a pivotal role is played by the PI3K/AKT–SGK1/mTOR signaling cascade [10, 105, 106].

Given the above, it is not surprising that all the non-specific antiviral drugs here described, i.e. the anticancer drugs repositionable in COVID-19 therapy deal with energy metabolism and inflammation.

A set of the drugs described here, e.g. those with explicit antiviral effect, can be preferred for the early stages of SARS-CoV-2 infection, while those dedicated to restraining the cytokine response – and without explicit antiviral effect - should be employed, if necessary, at later time points. Anyway, we should always consider that, even if the medications discussed in this review are safely in use in the clinics, the final decision for their administration in COVID-19 for compassionate and urgent use, when in the absence of validated clinical trials, should be taken solely after collegial approval by the clinical team taking care of the patient and under strict clinical surveillance. Indeed, unpredictable toxic side effects can arise, possibly linked with the patient clinical status or to the simultaneous administration of other drugs.

Finally, an interesting evaluation on how COVID-19 pandemic will affect the clinical care in the seven comprehensive cancer centers of Cancer Core Europe is discussed in a timely paper [107]. The authors illustrate appropriate guidelines that can transform this pandemic into an opportunity, e.g. for the assessment of the clinical effects of de-escalating anticancer regimens, forcedly imposed in order to prevent or reduce iatrogenic neutropenia and lymphopenia.

We hope that these findings may pave the way for a more comprehensive clinical experimentation on repurposing of ‘old’ drugs to the treatment of COVID-19, a line of research sustained by scant funds but of prime importance to face this new worldwide challenge.

## Data Availability

Not applicable.

## Abbreviations

BCG: Bacillus Calmette-Guérin
COVID-19: Coronavirus disease 2019
CQ: Chloroquine
EMT: Epithelial-to-mesenchymal transition
HCQ: Hydroxychloroquine
IFN: Interferon
MoAB: Monoclonal antibody
mTOR: Mammalian Target Of Rapamycin
SARS-CoV-2: Severe acute respiratory syndrome coronavirus 2
RA: Rheumatoid arthritis

## References

[1] Ashburn TT, Thor KB (2004). Drug repositioning: identifying and developing new uses for existing drugs. Nat Rev Drug Discov.

[2] Abbruzzese C, Matteoni S, Signore M, Cardone L, Nath K, Glickson JD (2017). Drug repurposing for the treatment of glioblastoma multiforme. J Exp Clin Cancer Res.

[3] Abbruzzese C, Matteoni S, Persico M, Villani V, Paggi MG (2020). Repurposing chlorpromazine in the treatment of glioblastoma multiforme: analysis of literature and forthcoming steps. J Exp Clin Cancer Res.

[4] Elfiky AA (2020). Anti-HCV, nucleotide inhibitors, repurposing against COVID-19. Life Sci.

[5] Zhou Y, Hou Y, Shen J, Huang Y, Martin W, Cheng F (2020). Network-based drug repurposing for novel coronavirus 2019-nCoV/SARS-CoV-2. Cell Discov.

[6] Baron SA, Devaux C, Colson P, Raoult D, Rolain JM. Teicoplanin: an alternative drug for the treatment of coronavirus COVID-19? Int J Antimicrob Agents. 2020;105944. 10.1016/j.ijantimicag.2020.105944. 10.1016/j.ijantimicag.2020.105944 PMC7102624 32179150

[7] Fan HH, Wang LQ, Liu WL, An XP, Liu ZD, He XQ, et al. Repurposing of clinically approved drugs for treatment of coronavirus disease 2019 in a 2019-novel coronavirus (2019-nCoV) related coronavirus model. Chin Med J. 2020. 10.1097/CM9.0000000000000797. 10.1097/CM9.0000000000000797 PMC7147283 32149769

[8] Li G, De Clercq E (2020). Therapeutic options for the 2019 novel coronavirus (2019-nCoV). Nat Rev Drug Discov.

[9] Chen YW, Yiu CB, Wong KY (2020). Prediction of the SARS-CoV-2 (2019-nCoV) 3C-like protease (3CL (pro)) structure: virtual screening reveals velpatasvir, ledipasvir, and other drug repurposing candidates. F1000Res.

[10] Dancey J (2010). mTOR signaling and drug development in cancer. Nat Rev Clin Oncol.

[11] Seto B (2012). Rapamycin and mTOR: a serendipitous discovery and implications for breast cancer. Clin Transl Med.

[12] Bride KL, Vincent T, Smith-Whitley K, Lambert MP, Bleesing JJ, Seif AE (2016). Sirolimus is effective in relapsed/refractory autoimmune cytopenias: results of a prospective multi-institutional trial. Blood..

[13] Copp J, Manning G, Hunter T (2009). TORC-specific phosphorylation of mammalian target of rapamycin (mTOR): phospho-Ser2481 is a marker for intact mTOR signaling complex 2. Cancer Res.

[14] Ling NXY, Kaczmarek A, Hoque A, Davie E, Ngoei KRW, Morrison KR (2020). mTORC1 directly inhibits AMPK to promote cell proliferation under nutrient stress. Nat Metab.

[15] Herzig S, Shaw RJ (2018). AMPK: guardian of metabolism and mitochondrial homeostasis. Nat Rev Mol Cell Biol.

[16] Valvezan AJ, Manning BD (2019). Molecular logic of mTORC1 signalling as a metabolic rheostat. Nature Metabolism..

[17] McNulty S, Flint M, Nichol ST, Spiropoulou CF (2013). Host mTORC1 signaling regulates Andes virus replication. J Virol.

[18] Stohr S, Costa R, Sandmann L, Westhaus S, Pfaender S, Anggakusuma (2016). Host cell mTORC1 is required for HCV RNA replication. Gut..

[19] Kindrachuk J, Ork B, Hart BJ, Mazur S, Holbrook MR, Frieman MB (2015). Antiviral potential of ERK/MAPK and PI3K/AKT/mTOR signaling modulation for Middle East respiratory syndrome coronavirus infection as identified by temporal kinome analysis. Antimicrob Agents Chemother.

[20] Wang CH, Chung FT, Lin SM, Huang SY, Chou CL, Lee KY (2014). Adjuvant treatment with a mammalian target of rapamycin inhibitor, sirolimus, and steroids improves outcomes in patients with severe H1N1 pneumonia and acute respiratory failure. Crit Care Med.

[21] Oza AM, Pignata S, Poveda A, McCormack M, Clamp A, Schwartz B (2015). Randomized phase II trial of Ridaforolimus in advanced endometrial carcinoma. J Clin Oncol.

[22] Klionsky DJ, Abdelmohsen K, Abe A, Abedin MJ, Abeliovich H, Acevedo Arozena A (2016). Guidelines for the use and interpretation of assays for monitoring autophagy (3rd edition). Autophagy..

[23] Wang Z, Liu P, Chen Q, Deng S, Liu X, Situ H (2016). Targeting AMPK signaling pathway to overcome drug resistance for Cancer therapy. Curr Drug Targets.

[24] Yoshida GJ (2015). Metabolic reprogramming: the emerging concept and associated therapeutic strategies. J Exp Clin Cancer Res.

[25] Vitale I, Manic G, Dandrea V, De Maria R (2015). Role of autophagy in the maintenance and function of cancer stem cells. Int J Dev Biol.

[26] Kimura T, Takabatake Y, Takahashi A, Isaka Y (2013). Chloroquine in cancer therapy: a double-edged sword of autophagy. Cancer Res.

[27] Mulcahy Levy JM, Towers CG, Thorburn A (2017). Targeting autophagy in cancer. Nat Rev Cancer.

[28] Pascolo S (2016). Time to use a dose of Chloroquine as an adjuvant to anti-cancer chemotherapies. Eur J Pharmacol.

[29] Briceno E, Calderon A, Sotelo J (2007). Institutional experience with chloroquine as an adjuvant to the therapy for glioblastoma multiforme. Surg Neurol.

[30] Sotelo J, Briceno E, Lopez-Gonzalez MA (2006). Adding chloroquine to conventional treatment for glioblastoma multiforme: a randomized, double-blind, placebo-controlled trial. Ann Intern Med.

[31] Briceno E, Reyes S, Sotelo J (2003). Therapy of glioblastoma multiforme improved by the antimutagenic chloroquine. Neurosurg Focus.

[32] Rolain JM, Colson P, Raoult D (2007). Recycling of chloroquine and its hydroxyl analogue to face bacterial, fungal and viral infections in the 21st century. Int J Antimicrob Agents.

[33] Savarino A, Boelaert JR, Cassone A, Majori G, Cauda R (2003). Effects of chloroquine on viral infections: an old drug against today's diseases?. Lancet Infect Dis.

[34] Olofsson S, Bergstrom T (2005). Glycoconjugate glycans as viral receptors. Ann Med.

[35] Devaux CA, Rolain JM, Colson P, Raoult D. New insights on the antiviral effects of chloroquine against coronavirus: what to expect for COVID-19? Int J Antimicrob Agents. 2020;105938. 10.1016/j.ijantimicag.2020.105938. 10.1016/j.ijantimicag.2020.105938 PMC7118659 32171740

[36] Walls AC, Park YJ, Tortorici MA, Wall A, McGuire AT, Veesler D. Structure, function, and antigenicity of the SARS-CoV-2 spike glycoprotein. Cell. 2020. 10.1016/j.cell.2020.02.058. 10.1016/j.cell.2020.02.058 PMC7102599 32155444

[37] Simmons G, Bertram S, Glowacka I, Steffen I, Chaipan C, Agudelo J (2011). Different host cell proteases activate the SARS-coronavirus spike-protein for cell-cell and virus-cell fusion. Virology..

[38] Kono M, Tatsumi K, Imai AM, Saito K, Kuriyama T, Shirasawa H (2008). Inhibition of human coronavirus 229E infection in human epithelial lung cells (L132) by chloroquine: involvement of p38 MAPK and ERK. Antivir Res.

[39] Gao J, Tian Z, Yang X (2020). Breakthrough: Chloroquine phosphate has shown apparent efficacy in treatment of COVID-19 associated pneumonia in clinical studies. Biosci Trends.

[40] Garulli B, Di Mario G, Sciaraffia E, Accapezzato D, Barnaba V, Castrucci MR (2013). Enhancement of T cell-mediated immune responses to whole inactivated influenza virus by chloroquine treatment in vivo. Vaccine..

[41] van den Borne BE, Dijkmans BA, de Rooij HH, le Cessie S, Verweij CL (1997). Chloroquine and hydroxychloroquine equally affect tumor necrosis factor-alpha, interleukin 6, and interferon-gamma production by peripheral blood mononuclear cells. J Rheumatol.

[42] Ye Z, Zhang Y, Wang Y, Huang Z, Song B. Chest CT manifestations of new coronavirus disease 2019 (COVID-19): a pictorial review. Eur Radiol. 2020. 10.1007/s00330-020-06801-0. 10.1007/s00330-020-06801-0 PMC7088323 32193638

[43] Borba MGS, Val FFA, Sampaio VS, Alexandre MAA, Melo GC, Brito M (2020). Effect of high vs low doses of Chloroquine Diphosphate as adjunctive therapy for patients hospitalized with severe acute respiratory syndrome coronavirus 2 (SARS-CoV-2) infection: a randomized clinical trial. JAMA Netw Open.

[44] Tang W, Cao Z, Han M, Wang Z, Chen J, Sun W, et al. Hydroxychloroquine in patients with COVID-19: an open-label, randomized, controlled trial. medRxiv. 2020:2020.04.10.20060558. 10.1101/2020.04.10.20060558.

[45] Magagnoli J, Narendran S, Pereira F, Cummings T, Hardin JW, Sutton SS, et al. Outcomes of hydroxychloroquine usage in United States veterans hospitalized with Covid-19. medRxiv. 2020:2020.04.16.20065920. 10.1101/2020.04.16.20065920. 10.1016/j.medj.2020.06.001 PMC7274588 32838355

[46] Saxton RA, Sabatini DM (2017). mTOR signaling in growth, metabolism, and disease. Cell..

[47] Sahraei Z, Shabani M, Shokouhi S, Saffaei A. Aminoquinolines against coronavirus disease 2019 (COVID-19): Chloroquine or Hydroxychloroquine. Int J Antimicrob Agents. 2020;105945. 10.1016/j.ijantimicag.2020.105945. 10.1016/j.ijantimicag.2020.105945 PMC7156117 32194152

[48] Rubin EJ, Baden LR, Morrissey S (2020). Audio interview: new research on possible treatments for Covid-19. N Engl J Med.

[49] Amato R, D'Antona L, Porciatti G, Agosti V, Menniti M, Rinaldo C (2009). Sgk1 activates MDM2-dependent p53 degradation and affects cell proliferation, survival, and differentiation. J Mol Med (Berl).

[50] Amato R, Scumaci D, D'Antona L, Iuliano R, Menniti M, Di Sanzo M (2013). Sgk1 enhances RANBP1 transcript levels and decreases taxol sensitivity in RKO colon carcinoma cells. Oncogene..

[51] D'Antona L, Amato R, Talarico C, Ortuso F, Menniti M, Dattilo V (2015). SI113, a specific inhibitor of the Sgk1 kinase activity that counteracts cancer cell proliferation. Cell Physiol Biochem.

[52] Talarico C, D'Antona L, Scumaci D, Barone A, Gigliotti F, Fiumara CV (2015). Preclinical model in HCC: the SGK1 kinase inhibitor SI113 blocks tumor progression in vitro and in vivo and synergizes with radiotherapy. Oncotarget.

[53] Abbruzzese C, Catalogna G, Gallo E, di Martino S, Mileo AM, Carosi M (2017). The small molecule SI113 synergizes with mitotic spindle poisons in arresting the growth of human glioblastoma multiforme. Oncotarget.

[54] Matteoni S, Abbruzzese C, Matarrese P, De Luca G, Mileo AM, Miccadei S (2019). The kinase inhibitor SI113 induces autophagy and synergizes with quinacrine in hindering the growth of human glioblastoma multiforme cells. J Exp Clin Cancer Res.

[55] Mahallawi WH, Khabour OF, Zhang Q, Makhdoum HM, Suliman BA (2018). MERS-CoV infection in humans is associated with a pro-inflammatory Th1 and Th17 cytokine profile. Cytokine..

[56] Wu C, Yosef N, Thalhamer T, Zhu C, Xiao S, Kishi Y (2013). Induction of pathogenic TH17 cells by inducible salt-sensing kinase SGK1. Nature..

[57] Spagnuolo R, Dattilo V, D'Antona L, Cosco C, Tallerico R, Ventura V, et al. Deregulation of SGK1 in ulcerative colitis: a paradoxical relationship between immune cells and colonic epithelial cells. Inflamm Bowel Dis. 2018. 10.1093/ibd/izy158. 10.1093/ibd/izy158 29788407

[58] Sebba A (2008). Tocilizumab: the first interleukin-6-receptor inhibitor. Am J Health Syst Pharm.

[59] Ishibashi K, Koguchi T, Matsuoka K, Onagi A, Tanji R, Takinami-Honda R (2018). Interleukin-6 induces drug resistance in renal cell carcinoma. Fukushima J Med Sci.

[60] Zhao L, Yang Y, Li W, Li T, Gao Q (2018). Nivolumab-induced cytokine-release syndrome in relapsed/refractory Hodgkin's lymphoma: a case report and literature review. Immunotherapy..

[61] Oved JH, Barrett DM, Teachey DT (2019). Cellular therapy: immune-related complications. Immunol Rev.

[62] Shimabukuro-Vornhagen A, Godel P, Subklewe M, Stemmler HJ, Schlosser HA, Schlaak M (2018). Cytokine release syndrome. J Immunother Cancer.

[63] Xu X, Han M, Li T, Sun W, Wang D, Fu B, et al. Effective treatment of severe COVID-19 patients with tocilizumab. Proc Natl Acad Sci U S A. 2020. 10.1073/pnas.2005615117. 10.1073/pnas.2005615117 PMC7245089 32350134

[64] Sperimentazioni cliniche - COVID-19. https://www.aifa.gov.it/sperimentazioni-cliniche-covid-19. Accessed Apr 21 2020.

[65] Tocilizumab in COVID-19 Pneumonia (TOCIVID-19). https://clinicaltrials.gov/ct2/show/NCT04317092. Accessed Apr 21 2020.

[66] Huizinga TW, Fleischmann RM, Jasson M, Radin AR, van Adelsberg J, Fiore S (2014). Sarilumab, a fully human monoclonal antibody against IL-6Ralpha in patients with rheumatoid arthritis and an inadequate response to methotrexate: efficacy and safety results from the randomised SARIL-RA-MOBILITY part a trial. Ann Rheum Dis.

[67] Kampan NC, Xiang SD, McNally OM, Stephens AN, Quinn MA, Plebanski M (2018). Immunotherapeutic Interleukin-6 or Interleukin-6 receptor blockade in Cancer: challenges and opportunities. Curr Med Chem.

[68] Al-Salama ZT (2019). Emapalumab: First Global Approval. Drugs..

[69] Lounder DT, Bin Q, de Min C, Jordan MB (2019). Treatment of refractory hemophagocytic lymphohistiocytosis with emapalumab despite severe concurrent infections. Blood Adv.

[70] Mertens M, Singh JA (2009). Anakinra for rheumatoid arthritis. Cochrane Database Syst Rev.

[71] Andre P, Denis C, Soulas C, Bourbon-Caillet C, Lopez J, Arnoux T (2018). Anti-NKG2A mAb is a checkpoint inhibitor that promotes anti-tumor immunity by unleashing both T and NK cells. Cell..

[72] Tinker AV, Hirte HW, Provencher D, Butler M, Ritter H, Tu D (2019). Dose-ranging and cohort-expansion study of Monalizumab (IPH2201) in patients with advanced gynecologic malignancies: a trial of the Canadian Cancer trials group (CCTG): IND221. Clin Cancer Res.

[73] Haanen JB, Cerundolo V (2018). NKG2A, a new kid on the immune checkpoint block. Cell..

[74] Zheng M, Gao Y, Wang G, Song G, Liu S, Sun D, et al. Functional exhaustion of antiviral lymphocytes in COVID-19 patients. Cell Mol Immunol. 2020. 10.1038/s41423-020-0402-2. 10.1038/s41423-020-0402-2 PMC7091858 32203188

[75] O'Mahony D, Kummar S, Gutierrez ME (2005). Non-small-cell lung cancer vaccine therapy: a concise review. J Clin Oncol.

[76] Usher NT, Chang S, Howard RS, Martinez A, Harrison LH, Santosham M (2019). Association of BCG vaccination in childhood with subsequent Cancer diagnoses: a 60-year follow-up of a clinical trial. JAMA Netw Open.

[77] Joice GA, Bivalacqua TJ, Kates M (2019). Optimizing pharmacokinetics of intravesical chemotherapy for bladder cancer. Nat Rev Urol.

[78] Freyne B, Marchant A, Curtis N (2015). BCG-associated heterologous immunity, a historical perspective: experimental models and immunological mechanisms. Trans R Soc Trop Med Hyg.

[79] Souza-Machado A, Cruz AA (2010). BCG vaccination and reduced risk of asthma. J Bras Pneumol.

[80] Tian X, Tian X, Huo R, Chang Q, Zheng G, Du Y (2017). Bacillus Calmette-Guerin alleviates airway inflammation and remodeling by preventing TGF-beta1 induced epithelial-mesenchymal transition. Hum Vaccin Immunother.

[81] Volpin F, Casaos J, Sesen J, Mangraviti A, Choi J, Gorelick N (2017). Use of an anti-viral drug, ribavirin, as an anti-glioblastoma therapeutic. Oncogene..

[82] Hadaczek P, Ozawa T, Soroceanu L, Yoshida Y, Matlaf L, Singer E (2013). Cidofovir: a novel antitumor agent for glioblastoma. Clin Cancer Res.

[83] Okubo K, Isono M, Asano T, Sato A (2019). Lopinavir-Ritonavir Combination Induces Endoplasmic Reticulum Stress and Kills Urological Cancer Cells. Anticancer Res.

[84] Hampson L, Maranga IO, Masinde MS, Oliver AW, Batman G, He X (2016). A single-arm, proof-of-concept trial of Lopimune (Lopinavir/ritonavir) as a treatment for HPV-related pre-invasive cervical disease. PLoS One.

[85] Chu CM, Cheng VC, Hung IF, Wong MM, Chan KH, Chan KS (2004). Role of lopinavir/ritonavir in the treatment of SARS: initial virological and clinical findings. Thorax..

[86] Cao B, Wang Y, Wen D, Liu W, Wang J, Fan G, et al. A trial of Lopinavir-ritonavir in adults hospitalized with severe Covid-19. N Engl J Med. 2020. 10.1056/NEJMoa2001282. 10.1056/NEJMoa2001282 PMC7121492 32187464

[87] Paeshuyse J, Dallmeier K, Neyts J (2011). Ribavirin for the treatment of chronic hepatitis C virus infection: a review of the proposed mechanisms of action. Curr Opin Virol.

[88] Schwoebel ED, Ho TH, Moore MS (2002). The mechanism of inhibition of ran-dependent nuclear transport by cellular ATP depletion. J Cell Biol.

[89] Casaos J, Gorelick NL, Huq S, Choi J, Xia Y, Serra R (2019). The use of ribavirin as an anticancer therapeutic: will it go viral?. Mol Cancer Ther.

[90] Mulangu S, Dodd LE, Davey RT, Tshiani Mbaya O, Proschan M, Mukadi D (2019). A randomized, controlled trial of Ebola virus disease therapeutics. N Engl J Med.

[91] Wang M, Cao R, Zhang L, Yang X, Liu J, Xu M (2020). Remdesivir and chloroquine effectively inhibit the recently emerged novel coronavirus (2019-nCoV) in vitro. Cell Res.

[92] de Wit E, Feldmann F, Cronin J, Jordan R, Okumura A, Thomas T, et al. Prophylactic and therapeutic remdesivir (GS-5734) treatment in the rhesus macaque model of MERS-CoV infection. Proc Natl Acad Sci U S A. 2020. 10.1073/pnas.1922083117. 10.1073/pnas.1922083117 PMC7104368 32054787

[93] Adaptive COVID-19 Treatment Trial (ACTT). https://clinicaltrials.gov/ct2/show/NCT04280705. Accessed Apr 21 2020.

[94] Grein J, Ohmagari N, Shin D, Diaz G, Asperges E, Castagna A, et al. Compassionate use of Remdesivir for patients with severe Covid-19. N Engl J Med. 2020. 10.1056/NEJMoa2007016. 10.1056/NEJMoa2007016 PMC7169476 32275812

[95] Wang Y, Zhang D, Du G, Du R, Zhao J, Jin Y, et al. Remdesivir in adults with severe COVID-19: a randomised, double-blind, placebo-controlled, multicentre trial. Lancet. 2020. 10.1016/S0140-6736(20)31022-9. 10.1016/S0140-6736(20)31022-9 PMC7190303 32423584

[96] NIH Clinical Trial Shows Remdesivir Accelerates Recovery from Advanced COVID-19. https://www.niaid.nih.gov/news-events/nih-clinical-trial-shows-remdesivir-accelerates-recovery-advanced-covid-19. Accessed 21 Apr 2020.

[97] Taubenberger JK, Kash JC, Morens DM. The 1918 influenza pandemic: 100 years of questions answered and unanswered. Sci Transl Med. 2019;11(502). 10.1126/scitranslmed.aau5485. 10.1126/scitranslmed.aau5485 PMC11000447 31341062

[98] Wan Y, Shang J, Graham R, Baric RS, Li F. Receptor Recognition by the Novel Coronavirus from Wuhan: an Analysis Based on Decade-Long Structural Studies of SARS Coronavirus. J Virol. 2020;94(7). 10.1128/JVI.00127-20. 10.1128/JVI.00127-20 PMC7081895 31996437

[99] Zhang L, Lin D, Sun X, Curth U, Drosten C, Sauerhering L, et al. Crystal structure of SARS-CoV-2 main protease provides a basis for design of improved alpha-ketoamide inhibitors. Science. 2020:eabb3405. 10.1126/science.abb3405. 10.1126/science.abb3405 PMC7164518 32198291

[100] Gunn BM, Roy V, Karim MM, Hartnett JN, Suscovich TJ, Goba A (2020). Survivors of Ebola virus disease develop Polyfunctional antibody responses. J Infect Dis.

[101] Ranadheera C, Coombs KM, Kobasa D (2018). Comprehending a killer: the Akt/mTOR signaling pathways are temporally high-jacked by the highly pathogenic 1918 influenza virus. EBioMedicine..

[102] Finlay DK (2015). Metabolic regulation of natural killer cells. Biochem Soc Trans.

[103] DeBerardinis RJ, Chandel NS (2020). We need to talk about the Warburg effect. Nat Metab.

[104] Epstein T, Gatenby RA, Brown JS (2017). The Warburg effect as an adaptation of cancer cells to rapid fluctuations in energy demand. PLoS One.

[105] Conciatori F, Ciuffreda L, Bazzichetto C, Falcone I, Pilotto S, Bria E, et al. mTOR Cross-Talk in Cancer and Potential for Combination Therapy. Cancers (Basel). 2018;10(1). 10.3390/cancers10010023. 10.3390/cancers10010023 PMC5789373 29351204

[106] Magaway C, Kim E, Jacinto E. Targeting mTOR and Metabolism in Cancer: Lessons and Innovations. Cells. 2019;8(12). 10.3390/cells8121584. 10.3390/cells8121584 PMC6952948 31817676

[107] van de Haar J, Hoes LR, Coles CE, Seamon K, Fröhling S, Jäger D, et al. Caring for patients with cancer in the COVID-19 era. Nat Med. 2020. 10.1038/s41591-020-0874-8. 10.1038/s41591-020-0874-8 32405058

